# Sex- and body mass index-specific reference intervals for serum leptin: a population based study in China

**DOI:** 10.1186/s12986-022-00689-x

**Published:** 2022-08-08

**Authors:** Jiayu Cheng, Yingying Luo, Yufeng Li, Fang Zhang, Xiuying Zhang, Xianghai Zhou, Linong Ji

**Affiliations:** 1 Department of Endocrinology and Metabolism, Peking University People’s Hospital, No. 11, Xizhimen South Street, Xicheng District, Beijing, 100044 China; 2 Department of Endocrinology and Metabolism, Capital Medical University Pinggu Teaching Hospital, No. 59, Xinping North Road, Pinggu District, Beijing, 101200 China

**Keywords:** Serum leptin, Reference intervals, Sex, BMI, Community population

## Abstract

**Background:**

Leptin is a peptide hormone secreted by adipose tissue and is an important determinant of obesity and its complications. The purpose of this study was to establish sex- and body mass index (BMI)-specific reference intervals for serum leptin in a Chinese population and investigate the factors influencing leptin concentrations.

**Methods:**

Fasting serum leptin levels were assayed in 469 men and 773 women from randomly sampled Chinese residents. Blood glucose, insulin, hemoglobin A1c (HbA1c), liver enzymes, blood lipid profiles, creatinine, and uric acid (UA) levels were measured. Pearson’s correlation coefficient and multiple linear regression analyses were used to estimate the relationship between serum leptin level and other variables. The reference intervals were determined by the 2.5th and 97.5th percentiles.

**Results:**

The mean ± standard deviation serum leptin level was much higher in women (20.92 ± 12.96 ng/mL) than in men (6.45 ± 5.53 ng/mL). The reference interval of serum leptin was 0.33–19.85 ng/mL in men and 3.60–54.86 ng/mL in women. The specific reference intervals of serum leptin in men with BMI of 20 to < 25 and 25 to < 27.5 kg/m^2^ were 0.42–12.32 and 2.17–20.22 ng/ml, respectively. The specific reference intervals of serum leptin in women with BMI of 20 to < 25 and 25 to < 27.5 kg/m^2^ were 4.11–38.09 and 8.27–48.66 ng/ml, respectively. BMI was significantly correlated with Ln (leptin) both in men (r = 0.698, *P* < 0.001) and women (r = 0.626, *P* < 0.001). In multivariate linear regression analysis, serum leptin was correlated with BMI, homeostasis model assessment of insulin resistance (HOMA-IR), UA in women, and plus triglyceride (TG) in men. The variance in serum leptin levels could be partially explained by these variables in both women (adjusted R^2^ = 0.447) and men (adjusted R^2^ = 0.552). In participants with leptin levels higher than the reference intervals, significantly higher levels of HOMA-IR, low-density lipoprotein cholesterol (LDL-C), UA, a higher proportion of central obesity (waist circumference [WC] > 90 cm), and metabolic syndrome were found in men, and significantly higher levels of HOMA-IR, UA and a higher proportion of central obesity (WC > 85 cm) were found in women.

**Conclusion:**

This is the first study to establish sex- and BMI-specific reference intervals of leptin for both sexes in a large Chinese population. Serum concentration of leptin was predicted by BMI, HOMA-IR, UA in women, and TG in men.

**Supplementary Information:**

The online version contains supplementary material available at 10.1186/s12986-022-00689-x.

## Introduction

Obesity and its’ comorbidities remain critical global public health issues. Over the past several decades, the number of obese women and men has increased to 390 (363–418) and 281 (257–307) million worldwide [1]. Leptin, with a size of 16 kDa, is a product of the obese (*ob*) gene [2]. This peptide hormone is mainly secreted by adipose tissue, and can cross the blood–brain barrier and act on neurons in the hypothalamus and elsewhere in the brain, thereby modulating food intake and body weight [3]. Under physiological conditions, the level of leptin increases when the mass of fat increases, thereby inhibiting food intake and forming a negative feedback loop [4]. In addition, leptin can regulate bone mass, reproductive function, and immune homeostasis [5]. High leptin levels are associated with greater risks of diabetes [6], non-alcoholic fatty liver disease [7], congestive heart failure, and cardiovascular disease [8]. Due to the lack of reference intervals for leptin in Chinese populations, studies on the association between leptin and the diseases described above have used different leptin cut-off points, which may be one of the reasons for the inconsistent results of these studies. Moreover, the terms “type 1 obesity” and “type 2 obesity,” which mean leptin hyposecretion or leptin resistance [4], need to be distinguished by determining the reference intervals of leptin.

Only a few studies have paid attention to the reference intervals of leptin levels in Asian healthy adults [9, 10]. Moreover, serum concentrations of leptin reflect the amount of energy stored in adipose tissue and may vary in between sexes and individuals with different BMI [11]. Although a small-scale study proposed reference intervals for leptin in native Chinese women, it did not include male participants or determine the BMI-specific reference intervals for leptin [9].

In this study, we aimed to establish sex- and BMI-specific reference intervals for serum leptin in a randomly sampled cohort from residents of Pinggu District in Beijing, as well as to identify the factors correlated with serum leptin concentrations.

## Materials and methods

### Study population

**Fig. 1:**
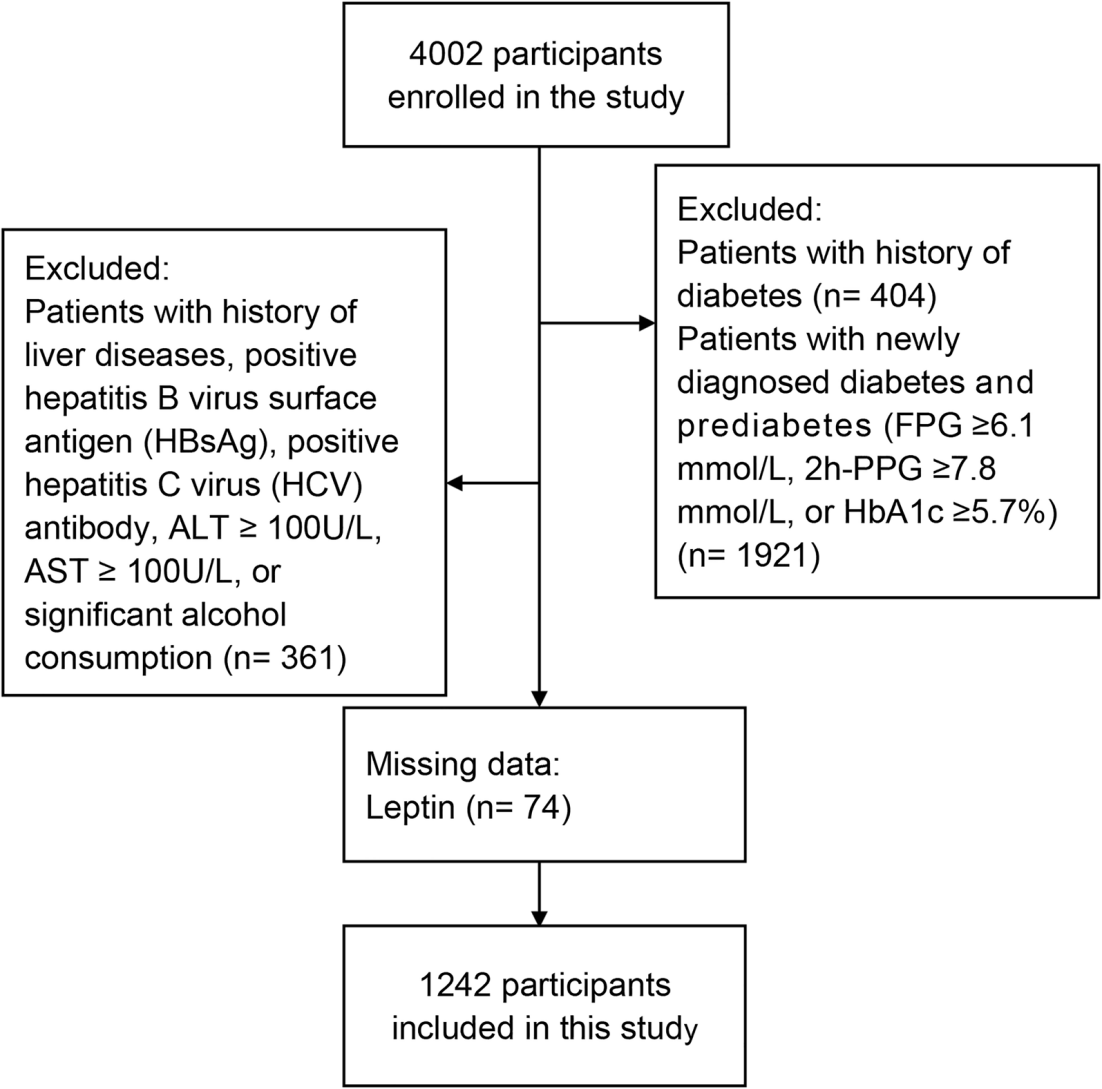
Flow diagram for the study population

This is a cross-sectional study. Details of participant selection have been published in our previous publication [12]. Briefly, from September 2013 to July 2014, 6,583 individuals aged 25 to 76 years old living in the Pinggu District of Beijing, China were invited to participate in the Pinggu metabolic disease study using two-stage cluster random sampling and 4,002 individuals participated in the study. In the current analysis, a healthy population was defined by excluding participants with the following conditions (Fig. 1): (a) a history of diabetes (n = 404); (b) newly diagnosed diabetes and prediabetes (fasting plasma glucose [FPG] ≥ 6.1 mmol/L, and/or 2-h postprandial plasma glucose (PPG) after a 75-g oral glucose tolerance test [2 h-PPG] ≥ 7.8 and/or hemoglobin A1c [HbA1c] ≥ 5.7%;n = 1921) [13]; (c) self-reported history of liver diseases, including positive hepatitis B virus surface antigen (HBsAg), positive hepatitis C virus (HCV) antibody, alanine aminotransferase (ALT) ≥ 100 U/L, aspartate aminotransferase (AST) ≥ 100 U/L, or significant alcohol consumption (men > 210 g/week or women > 140 g/week [13]) (n = 361). Finally, after excluding participants with missing data on leptin concentrations (n = 74), the data of 1,242 healthy individuals were used to determine the reference intervals and correlated factors of leptin level. The study protocol was approved by the ethics committees of the Peking University Medical Center and the University of Michigan. Written informed consent was obtained from all the participants.

### Variables collection

Participant characteristics and medical histories were recorded through personal interviews with trained doctors and nurses. Weight and height were measured in light clothing, and BMI was calculated as weight divided by height in meters squared (kg/m^2^). Waist circumference (WC) was measured at the midpoint of the line between the lower edge of the ribs and iliac crest, and hip circumference (HC) at the level of the greater trochanter. After 10 min of rest, blood pressure was measured three times, and the mean systolic blood pressure (SBP) and diastolic blood pressure (DBP) were used. Participants reported their alcohol intake frequency and volume of drinks to determine alcohol consumption (g/week).

Venous blood samples were collected after 10–12 h of fasting. A standard 75-g glucose tolerance test was performed in individuals without previously diagnosed diabetes. Plasma glucose, total cholesterol (TC), triglycerides (TG), high-density lipoprotein cholesterol (HDL-C), low-density lipoprotein cholesterol (LDL-C), ALT, AST, serum creatinine (Cr), and uric acid (UA) levels were measured using an automated routine laboratory analyzer (UnicelDxC 800; Beckman Coulter, USA). HbA1c levels were measured by high-performance liquid chromatography (Adams A1c HA-8160; Arkray, Japan). Insulin was assayed by electrochemiluminescence (Roche E411; Roche Diagnostics). The severity of insulin resistance was evaluated by the homeostasis model assessment of insulin resistance (HOMA-IR), which was calculated as follows: HOMA-IR = FPG (mmol/L) × fasting insulin (μU/mL)/22.5 [14]. Serum leptin level was measured using a commercial ELISA kit (EMDMilipol, Billerica, MA, USA). The intra-assay coefficient variation of the ELISA method is < 5%, and the inter-assay coefficient variation is < 8.6%. HBsAg and HCV antibodies were measured by enhanced chemiluminescence assays (Ortho-Clinical Diagnostics, NJ, USA). According to the international diabetes federation (IDF) criteria, metabolic syndrome (MetS) is diagnosed if there is central obesity (WC > 90 cm for men, and > 85 cm for women, respectively), in addition to any two of the following four criteria: (1) TG level ≥ 1.7 mmol/L; (2) HDL-C < 1.0 mmol/L for men or < 1.3 mmol/L for women; (3) BP ≥ 130/85 mmHg or treatment of previously diagnosed hypertension; and (4) FPG ≥ 5.6 mmol/L or previously diagnosed as type 2 diabetes [15].

### Statistical analysis

Separate analyses were conducted for men and women due to sex differences in serum leptin levels. Continuous variables with normal distribution are expressed as mean ± standard deviation (SD), and* t* test was used to compare differences between men and women. Continuous variables with skewed distribution were expressed as median (25th and 75th percentiles) and compared using the Mann–Whitney test. Leptin level was logarithmically transformed to Ln (leptin), because of the skewed distribution of serum leptin concentrations. Multiple linear regression analysis was used to estimate the association between the participant characteristics and serum leptin levels. Based on the Pearson’s correlation coefficients, variables with statistical significance and clinical significance were put into the multivariate linear regression model. In the multivariate linear regression analysis, variables were screened out stepwise to reach the largest adjusted R^2^. The adjusted R^2^ reflected the proportion of variation in leptin levels explained by participants’ characteristics. The sex- and BMI-specific reference intervals of serum leptin concentrations were determined using the 2.5th and 97.5th percentiles. In the supplementary files, Pearson’s correlation coefficients were used to assess the correlation between Ln (leptin) and the other variables. Participants were divided into three groups based on the 2.5th and 97.5th percentiles, and the tertiles of serum leptin levels, respectively. In addition, the differences in continuous variables among groups were compared using the analysis of variance or the Kruskal–Wallis test, and the differences in categorical variables among groups were compared using the Chi-square or Fisher’s exact tests. Receiver operating characteristic (ROC) analysis was performed for determining cut-off point of serum leptin levels, and the cut-off point was used to distinguish the participants with BMI < 25 kg/m^2^ (normal weight) and the participants with BMI ≥ 25 kg/m^2^ (overweight or obesity). Statistical significance was set at *P* < 0.05. The IBM SPSS Statistics software (version 21.0) was used for the statistical analysis.

## Results

A total of 1242 participants were included in the study (Table 1). The mean age of the 1242 participants was 44.8 ± 11.6 years, and 37.8% (469) were men. The average BMI of the participants was 24.8 ± 3.6 kg/m^2^. The male participants were older than the female participants (46.1 ± 12.6 vs. 43.9 ± 10.8 years). Men were likely to be more frequent smokers, and have a higher proportion of metabolic syndrome, higher levels of BMI, WC, HC, blood pressure, FPG, TG, and serum UA than women. In contrast, women showed higher 2 h-PPG, HDL-C, and serum leptin levels than men. There were no significant differences in HOMA-IR, HbA1c, TC concentration, and LDL-C concentration between the two sexes. The median (25th and 75th percentiles) serum leptin was 12.95 (5.96 and 21.40) ng/mL in the overall population. Women had higher leptin levels (17.90 ng/mL) than men (5.20 ng/mL).

**Table 1:** Clinical characteristics of the study participants

	Total	Men	Women	*P* value*
N	1242	469	773	–
Age, y	44.8 ± 11.6	46.1 ± 12.6	43.9 ± 10.8	0.002
Smoking, n (%)	310 (25.0)	298 (63.5)	12 (1.6)	< 0.001
BMI, kg/m^2^	24.8 ± 3.6	25.1 ± 3.6	24.6 ± 3.5	0.029
WC, cm	81.7 ± 9.8	85.7 ± 9.9	79.3 ± 9.0	< 0.001
HC, cm	96.6 ± 6.6	97.3 ± 6.6	96.3 ± 6.5	0.009
SBP, mmHg	123.2 ± 17.0	127.2 ± 15.2	120.8 ± 17.5	< 0.001
DBP, mmHg	75.4 ± 10.6	77.9 ± 10.6	73.8 ± 10.4	< 0.001
FPG, mmol/L	5.3 ± 0.4	5.4 ± 0.3	5.2 ± 0.4	< 0.001
HOMA-IR, median (25th, 75th)	1.68 (1.15, 2.33)	1.69 (1.15, 2.39)	1.68 (1.16, 2.30)	0.481
2 h-PPG, mmol/L	6.0 ± 1.0	5.8 ± 1.1	6.1 ± 1.0	< 0.001
HbA1c, %	5.3 ± 0.2	5.4 ± 0.2	5.3 ± 0.2	0.334
TC, mmol/L	4.66 ± 0.85	4.64 ± 0.83	4.68 ± 0.86	0.375
TG, mmol/L	1.20 ± 0.96	1.46 ± 1.11	1.04 ± 0.82	< 0.001
TG, median (25th, 75th), mmol/L	0.96 (0.62, 1.44)	1.15 (0.78, 1.75)	0.85 (0.56, 1.28)	< 0.001
HDL-C, mmol/L	1.19 ± 0.30	1.08 ± 0.27	1.26 ± 0.30	< 0.001
LDL-C, mmol/L	2.74 ± 0.74	2.77 ± 0.74	2.72 ± 0.73	0.272
ALT, U/L	20 ± 10	24 ± 12	18 ± 8	< 0.001
AST, U/L	21 ± 6	22 ± 6	20 ± 6	< 0.001
Serum Cr, μmol/L	58 ± 13	69 ± 11	51 ± 8	< 0.001
UA, μmol/L	266 ± 75	319 ± 74	233 ± 54	< 0.001
MetS, n (%)	269 (21.7)	169 (36.0)	100 (12.9)	< 0.001
Leptin, ng/ml	15.46 ± 12.85	6.45 ± 5.53	20.92 ± 12.96	< 0.001
Leptin, median (25th, 75th), ng/ml	12.49 (5.96, 21.40)	5.20 (2.62, 8.65)	17.90 (11.87, 26.88)	< 0.001

Continuous variables were expressed as mean ± standard deviation or median (25%th, 75%th), and categorical variables were expressed as n (%)

*P showed the comparison between men and women

Additional file 1: Table S1 shows the correlation between Ln (leptin) and the different factors in men and women. HOMA-IR was positively correlated with Ln (leptin), whereas age and HDL-C were negatively correlated with Ln (leptin) in both sexes. Notably, the correlation coefficients of TG and UA in men were likely to be higher than those in women. BMI was the strongest factor correlated with Ln (leptin) in both men (r = 0.698) and women (r = 0.626) (Additional file 1: Table S1).

Multivariate linear regression analysis identified the most relevant factors that may affect leptin concentration. In men, BMI, HOMA-IR, TG, and UA were the variables that strongly predicted Ln (leptin), and the calculation of predicted serum leptin concentration in men was as follows: Ln (leptin) = -3.039 + 0.142 × (BMI) + 0.189 × (HOMA-IR) + 0.074 (TG) + 0.002 × (UA) (Table 2). In contrast, BMI, HOMA-IR, and UA were the variables that strongly predicted Ln (leptin) in women, with the calculation for predicted serum leptin concentration being as follows: Ln (leptin) = 0.066 + 0.092 × (BMI) + 0.157 × (HOMA-IR) + 0.001 × (UA) (Table 2).

**Table 2:** Multiple linear regression of Ln (leptin) in each gender*

Variable	Men			Women		
	β	SE	*P* value	β	SE	*P* value
Intercept	− 3.039	–	–	0.066	–	–
BMI	0.142	0.010	< 0.001	0.092	0.006	< 0.001
HOMA-IR	0.189	0.034	< 0.001	0.157	0.019	< 0.001
TG	0.074	0.030	0.014	–	–	–
UA	0.002	< 0.001	0.001	0.001	< 0.001	0.004

*The adjusted R^2^ in men was 0.552; The adjusted R^2^ in women was 0.447

Table 3 shows the serum leptin concentrations in each sex according to age, WC, HOMA-IR, and UA subgroups. The serum leptin levels of participants with HOMA-IR ≥ 1.00 were significantly higher than that of participants with HOMA-IR < 1.00 in both women and men (*P* < 0.001). Besides, serum leptin levels were significantly higher in the high-UA subgroup than in the low-UA subgroup in both women (*P* = 0.004) and men (*P* < 0.001).

**Table 3:** Serum leptin concentrations (ng/ml) in each gender by different subgroups

	Men			Women		
	N	Median (25th,75th)	*P* value	N	Median (25th,75th)	*P* value
Overall	469	5.20 (2.62, 8.65)		773	17.90 (11.87, 26.88)	
*Age, y*			0.001			0.021
< 45	229	5.94 (3.05, 10.06)		428	19.20 (12.36, 28.29)	
45- < 65	205	4.54 (2.51, 7.11)		309	17.08 (11.56, 25.37)	
≥ 65	35	5.17 (1.27, 8.72)		36	13.92 (8.47, 24.06)	
WC, cm*			< 0.001			< 0.001
< 90/85	303	3.62 (1.85, 5.79)		567	15.64 (10.07, 22.87)	
≥ 90/85	166	8.60 (6.38, 12.52)		206	27.11 (19.25,37.81)	
*HOMA-IR*			< 0.001			< 0.001
< 1.00	79	1.91 (0.72, 3.24)		125	9.73 (6.94, 14.72)	
≥ 1.00	390	5.94 (3.62, 9.61)		648	20.17 (13.88, 28.62)	
*UA, mmol/L*			< 0.001			0.004
< 420	430	4.71 (2.49, 8.25)		768	17.84 (11.84, 26.75)	
≥ 420	39	8.33 (5.45, 13.75)		5	45.98 (25.81, 71.68)	

*P* value showed the comparison between different subgroups in each gender, respectively

*The cut-off point of WC is 90 cm for men, and 85 cm for women

Based on BMI levels, all participants were further divided into five subgroups: < 20, 20 to < 25, 25 to < 27.5, 27.5 to < 30, and ≥ 30 kg/m^2^. Table 4 shows the mean ± SD and reference intervals (2.5th and 97.5th percentiles) of the different BMI subgroups in each sex. Generally, the serum leptin levels of each BMI subgroup in women were much higher than those in men.

**Table 4:** Reference intervals of serum leptin (ng/ml) in each gender by BMI

Sex and BMI (kg/m^2^)	Mean ± SD	2.5th, 97.5th	25th	50th	75th
*Men*					
All BMI (n = 469)	6.45 ± 5.53	0.33, 19.85	2.62	5.20	8.65
< 20 (n = 34)	1.26 ± 1.10	0.16, −*	0.34	0.90	1.86
20 to < 25 (n = 198)	4.08 ± 3.14	0.42, 12.32	1.90	3.26	5.44
25 to < 27.5 (n = 127)	7.29 ± 5.73	2.17, 20.22	4.31	6.14	8.61
27.5 to < 30 (n = 66)	9.32 ± 3.93	2.86, 19.41	6.36	8.98	11.50
≥ 30 (n = 44)	14.42 ± 6.33	4.50, 30.20	8.98	13.66	18.18
*Women*					
All BMI (n = 773)	20.92 ± 12.96	3.60, 54.86	11.87	17.90	26.88
< 20 (n = 53)	9.23 ± 5.61	1.04, 22.74	4.78	8.34	12.74
20 to < 25 (n = 403)	16.29 ± 8.96	4.11, 38.09	9.96	14.92	20.53
25 to < 27.5 (n = 172)	24.27 ± 11.01	8.27, 48.66	16.33	22.24	31.47
27.5 to < 30 (n = 85)	29.31 ± 11.47	13.71, 62.54	21.01	26.20	36.20
≥ 30 (n = 60)	40.85 ± 16.62	14.32, 80.96	27.75	40.09	51.50

*We didn’t get the 97.5th of leptin concentrations due to the number limitation of participants in this subgroup

After determining the 2.5th and 97.5th percentiles for the serum leptin levels, we divided men and women with BMI of 20 to < 25 into three groups: leptin < 2.5th, 2.5th to ≤ 97.5th, and > 97.5th percentiles, respectively (Additional file 1: Table S2 and Additional file 1: Table S3). In men with a BMI of 20 to < 25, participants with leptin > 97.5th group had significantly higher levels of HOMA-IR, LDL-C, UA, a higher proportion of central obesity (WC > 90 cm), metabolic syndrome, and lower levels of HDL-C than participants with leptin of 2.5th to ≤ 97.5th group (Additional file 1: Table S2). Among women with a BMI of 20 to < 25, participants with leptin > 97.5th had significantly higher levels of HOMA-IR, UA and a higher proportion of central obesity (WC > 85 cm) compared to participants with leptin levels of 2.5th to ≤ 97.5th (Additional file 1: Table S3). In addition, as shown in Additional file 1: Table S3, participants with leptin levels > 97.5th seemed to have higher levels of LDL-C, and lower levels of HDL-C.

The clinical characteristics of men with BMI between 20 and 25 by tertiles of leptin levels are shown in Additional file 1: Table S4, and the clinical characteristics of women with BMI between 20 and 25 by tertiles of leptin levels are shown in Additional file 1: Table S5. ROC analysis for determining cut-off point of serum leptin levels is shown in Additional file 1: Table S6, along with the sensitivity, specificity, and the area under curve (AUC).

## Discussion

In this large population-based study, we analyzed the correlation of leptin with other clinical characteristics and biochemical factors, including age, BMI, WC, blood glucose, HOMA-IR, blood lipid profiles, and UA. Furthermore, we proposed formulas for serum leptin concentrations using multivariate linear regression analysis in both men and women. We established sex- and BMI-specific reference intervals for serum leptin levels in both sexes, based on a healthy Chinese population.

Previous studies have demonstrated a relationship between leptin levels and body fat distribution in various ethnic populations. A cross-sectional study found high correlations between leptin levels and BMI (r = 0.80 in men, 0.79 in women), and concluded that leptin production was proportional to adipose tissue mass [16]. Furthermore, another study indicated that leptin concentrations were significantly correlated with BMI (r = 0.741 in men, r = 0.814 in women) and WC (r = 0.840 in men, r = 0.718 in women) in Mexican Americans, and concluded a high association between leptin levels and overall adipose tissue depots rather than with a specific fat depot [17]. Our study found a similar correlation between serum leptin concentrations with both BMI (r = 0.698 in men and r = 0.626 in women) and WC (r = 0.712 in men and r = 0.554 in women) for both sexes. However, some studies have reported a significant correlation between leptin with other variables of body adiposity, such as skinfold thickness [18], percentage body fat [19] and truncal body fat [20].

In addition to physical measures, we investigated the association between leptin and biochemical variables. Our study showed a correlation between serum leptin concentrations and HOMA-IR in a Chinese population, which is consistent with the data of a previous study, indicating that leptin levels were positively associated with HOMA-IR (*P* < 0.001) in South Asian, Chinese, Aboriginal, and European Canadians [21]. Interestingly, a previous study showed a genetic correlation (r = 0.4785) and a shared genetic locus between circulating leptin concentrations and HOMA-IR [22]. Obesity leads to increased leptin levels and insulin resistance [23], and an experimental study indicated that leptin regulates the development of insulin resistance through its effects on the liver [24]. Elevated serum TG levels may lead to the expansion of adipose tissue, which may further increase serum leptin concentrations. In this study, we found that serum leptin levels were positively correlated with TG concentrations. This finding is consistent with the data of a large population-based study in Caucasian individuals, showing a significant correlation between leptin and TG:0.32 in women (*P* < 0.001) and 0.28 in men (*P* < 0.001) [25]. In addition, animal studies have suggested that leptin may influence serum triglyceride levels indirectly [26, 27].

Based on the significant correlation of leptin with BMI, HOMA-IR, TG, and UA, we proposed the predicted formulas for serum leptin levels for each sex, and the adjusted R^2^ of the formula was 0.552 and 0.447 for men and women, respectively. The multiple linear regression models in our study explained the degree of variance in serum leptin levels, as high as that explained by other models, including percentage body fat (R^2^ = 0.523 for men; R^2^ = 0.551 for women) estimated by the bioimpedance method [28] or total abdominal fat (R^2^ = 0.493 for men) assessed by abdominal CT [29].

To the best of our knowledge, our study is the first to establish sex- and BMI-specific reference intervals for leptin for both sexes in the largest Chinese cohort. In addition, this cohort was a random sampling population instead of the hospital population. A previous Chinese study investigating the reference values of serum leptin in women indicated that the mean ± SD value of serum leptin concentrations was 10.5 ± 1.99 ng/ml [9], which was significantly lower than the serum levels of leptin (20.92 ± 12.96 ng/mL) in women in our study [9]. The lower BMI levels (22.69 ± 3.26 kg/m^2^) observed in the above study [9] compared to that of our study (24.6 ± 3.5 kg/m^2^) may account for the variance of leptin concentrations. In addition, we established sex- and BMI-specific reference intervals for serum leptin concentrations in both sexes. A study of the data from the third National Health and Nutrition Examination Survey (NHANES III) showed the serum leptin concentrations by ethnicity and BMI in both genders [18], which had similar levels of leptin in men of all BMI groups (6.0 ± 0.16 ng/mL) as observed in our study. However, the serum leptin levels of all BMIs, < 20, 20 to < 25, 25 to < 27.5, 27.5 to < 30, and > 30 groups in women in our study seemed to be higher than that in the NHANES III study [18]. Another study in multiple ethnicities also found that compared with European women, South Asian women had strikingly high leptin concentrations, which could not be explained by fat distribution and hyperinsulinism [21]. However, there is evidence that hypertrophy of subcutaneous adipocytes may contribute to high leptin concentrations in South Asians [30].

After determining the reference interval of leptin levels, our further analysis showed that, in the normal BMI participants, individuals with leptin levels higher than the 97.5th percentile may have a higher risk of prevalent metabolic syndrome and abdominal obesity. This finding contributes to the clinical application of serum leptin measurement, but future studies with larger sample sizes are needed to support this finding.

The established reference intervals in our study provide reliable evidence for the clinical analysis and understanding of leptin, as well as a theoretical basis for the clinical application of leptin. However, our study had some limitations. First, the reference intervals for serum leptin concentrations can only reflect healthy individuals in the Han population. Second, we did not assess other specific fat distributions, such as skinfold thickness and visceral fat. Third, after excluding the participants who meet the exclusion criteria, there are differences in the number of participants with different age and sex. We excluded the participants with diabetes, so the higher prevalence of diabetes in men than in women may contribute to the differences. Of note, the sample size in our study is larger than previous study [9]. In addition, the median (25th, 75th) of serum leptin absolute levels with different ages are similar (Table 3).

## Conclusion

In conclusion, we established sex- and BMI-specific reference intervals for serum leptin concentrations for both sexes in a large Chinese cohort living in Pinggu area and examined the possible factors correlated with leptin.

## Supplementary Information

**Additional file 1**: **Table S1**. Correlation between Ln (leptin) and different variables in each gender. **Table S2**. Clinical characteristics of men with BMI of 20 to <25. **Table S3**. Clinical characteristics of women with BMI of 20 to <25. **Table S4**. Clinical characteristics of men with BMI of 20 to <25 by tertiles of leptin levels. **Table S5**. Clinical characteristics of women with BMI of 20 to <25 by tertiles of leptin levels. **Table S6**. ROC analysis for determining cut-off point of serum leptin levels (ng/ml) to distinguish the participants with BMI <25 kg/m^2^ (normal weight) and BMI ≥25 kg/m^2^ (overweight or obesity).

## Data Availability

All data generated or analyzed during this study are included in the article. Further inquiries can be directed to the corresponding author.

## Abbreviations

BMI: Body mass index
WC: Waist circumference
HC: Hip circumference
SBP: Systolic blood pressure
DBP: Diastolic blood pressure
FPG: Fasting plasma glucose
HOMA-IR: Homeostasis model assessment of insulin resistance
2 h-PPG: 2-Hour postprandial plasma glucose
HbA1c: Hemoglobin A1c
TC: Total cholesterol
TG: Triglyceride
HDL-C: High density lipoprotein cholesterol
LDL-C: Low density lipoprotein cholesterol
ALT: Alanine aminotransferase
AST: Aspartate aminotransferase
Cr: Creatinine
UA: Uric acid
MetS: Metabolic syndrome
