# Supplementary material for: Sex- and body mass index-specific reference intervals for serum leptin: a population based study in China
Source: Nutr Metab (Lond). 2022 Aug 8;19:54. doi: 10.1186/s12986-022-00689-x (PMC9358897; doi:10.1186/s12986-022-00689-x)
Supplement: Supplementary file 1 — Additional file 1: Table S1. Correlation between Ln (leptin) and different variables in each gender. Table S2. Clinical characteristics of men with BMI of 20 to <25. Table S3. Clinical characteristics of women with BMI of 20 to <25. Table S4. Clinical characteristics of men with BMI of 20 to <25 by tertiles of leptin levels. Table S5. Clinical characteristics of women with BMI of 20 to <25 by tertiles of leptin levels. Table S6. ROC analysis for determining cut-off point of serum leptin levels (ng/ml) to distinguish the participants with BMI <25 kg/m2 (normal weight) and BMI ≥25 kg/m2 (overweight or obesity). [file 12986_2022_689_MOESM1_ESM.docx]

**Supplementary Tables**

**Table S1** Correlation between Ln (leptin) and different variables in each gender

|  | **Men** |  | **Women** |  |
| --- | --- | --- | --- | --- |
|  | **Coefficient (r)** | ***P* value** | **coefficient (r)** | ***P* value** |
| Age | -0.192 | <0.001 | -0.102 | 0.004 |
| BMI | 0.698 | <0.001 | 0.626 | <0.001 |
| WC | 0.712 | <0.001 | 0.554 | <0.001 |
| HC | 0.592 | <0.001 | 0.548 | <0.001 |
| SBP | 0.070 | 0.129 | 0.045 | 0.207 |
| DBP | 0.181 | <0.001 | 0.174 | <0.001 |
| FPG | 0.117 | 0.011 | 0.154 | <0.001 |
| HOMA-IR* | 0.649 | <0.001 | 0.570 | <0.001 |
| 2h-PPG | 0.216 | <0.001 | 0.170 | <0.001 |
| HbA1c | 0.077 | 0.096 | 0.106 | 0.003 |
| TC | 0.121 | 0.008 | 0.070 | 0.053 |
| TG* | 0.403 | <0.001 | 0.304 | <0.001 |
| HDL-C | -0.379 | <0.001 | -0.194 | <0.001 |
| LDL-C | 0.163 | <0.001 | 0.126 | <0.001 |
| ALT | 0.320 | <0.001 | 0.138 | <0.001 |
| AST | 0.087 | 0.060 | -0.021 | 0.553 |
| Serum Cr | 0.201 | <0.001 | 0.138 | <0.001 |
| UA | 0.348 | <0.001 | 0.260 | <0.001 |

Pearson’s correlation analysis was used to assess the correlation of Ln (leptin) and other variables.

* Due to the skewed distribution of HOMA-IR and TG, the correlation coefficient was calculated after transforming logarithmically.

Abbreviation: BMI, body mass index; WC, waist circumference; HC, hip circumference; SBP, systolic blood pressure; DBP, diastolic blood pressure; FPG, fasting plasma glucose; HOMA-IR, homeostasis model assessment of insulin resistance; 2h-PPG, 2-hour postprandial plasma glucose; HbA1c, hemoglobin A1c; TC, total cholesterol; TG, triglyceride; HDL-C, high density lipoprotein cholesterol; LDL-C, low density lipoprotein cholesterol; ALT, alanine aminotransferase; AST, aspartate aminotransferase; Cr, creatinine; UA, urine acid.

**Table S2** Clinical characteristics of men with BMI of 20 to <25

|  | **Leptin <2.5^th^ (0.42 ng/ml)** | **Leptin of 2.5^th^ to ≤97.5^th^** | **Leptin >97.5^th^**  **(12.32 ng/ml)** | ***P* value** |
| --- | --- | --- | --- | --- |
| N | 12 | 624 | 19 |  |
| Age, y | 54.3±4.7 | 51.9±11.8 | 52.5±14.8 | 0.780 |
| Smoking, n (%)* | 11 (91.7) | 429 (68.8) | 7 (36.8) | 0.003 |
| Hypertension, n (%) | 0 (0) | 133 (21.3) | 5 (26.3) | 0.264 |
| Diabetes, n (%) | 0 (0) | 48 (7.7) | 3 (15.8) | 0.257 |
| Dyslipidemia, n (%) | 0 (0) | 82 (13.1) | 4 (21.1) | 0.438 |
| BMI, kg/m^2^* | 21.9±1.2 | 23.0±1.3 | 23.8±1.3 | 0.001 |
| WC, cm* | 75.4±3.0 | 81.2±5.7 | 85.9±6.3 | <0.001 |
| WC ≥90cm, n (%)* | 0 (0) | 41 (6.6) | 5 (26.3) | 0.003 |
| HC, cm* | 90.2±4.1 | 93.9±4.9 | 95.1±4.4 | 0.017 |
| SBP, mmHg | 118.9±12.4 | 130.9±17.7 | 129.0±14.5 | 0.060 |
| DBP, mmHg | 71.6±7.9 | 78.1±10.9 | 80.1±7.1 | 0.084 |
| FPG, mmol/L | 5.5±0.6 | 6.0±1.7 | 6.0±1.4 | 0.553 |
| HOMA-IR, median (25^th^, 75^th^)* | 0.63 (0.47, 0.89) | 1.31 (0.96, 1.98) | 2.51 (1.39, 3.24) | <0.001 |
| 2h-PPG, mmol/L | 5.6±1.7 | 6.9±2.6 | 7.2±1.6 | 0.201 |
| HbA1c, % | 5.3±0.3 | 5.7±1.0 | 5.6±0.5 | 0.395 |
| TC, mmol/L | 4.50±0.70 | 4.85±0.95 | 5.10±0.86 | 0.215 |
| TG, mmol/L | 0.80±0.44 | 1.33±1.27 | 1.50±0.81 | 0.290 |
| HDL-C, mmol/L* | 1.55±0.33 | 1.22±0.36 | 1.18±0.26 | 0.008 |
| LDL-C, mmol/L* | 2.16±0.52 | 2.80±0.76 | 3.11±0.71 | 0.003 |
| ALT, U/L* | 34.8±35.3 | 21.7±9.7 | 21.0±7.5 | <0.001 |
| AST, U/L* | 33.2±20.8 | 23.4±10.5 | 23.7±8.4 | 0.008 |
| Serum Cr, μmol/L* | 61.0±10.4 | 68.1±11.3 | 75.4±13.6 | 0.002 |
| UA, μmol/L* | 266.6±73.1 | 301.2±69.8 | 337.5±55.2 | 0.018 |
| MetS, n (%)* | 1 (8.3) | 238 (38.1) | 11 (57.9) | 0.022 |
| Leptin, ng/ml* | 0.30±0.06 | 3.78±2.70 | 15.58±4.83 | <0.001 |

Continuous variables were expressed as mean ± standard deviation or median (25%th, 75%th), and categorical variables were expressed as n (%).

* P values of the difference between the leptin >97.5^th^ group and the leptin of 2.5^th^ to ≤97.5^th^ group, and the difference between the leptin >97.5^th^ group and the leptin <2.5^th^ group were <0.025.

**Table S3** Clinical characteristics of women with BMI of 20 to <25

|  | **Leptin <2.5^th^ (4.11 ng/ml)** | **Leptin of 2.5^th^ to ≤97.5^th^** | **Leptin >97.5^th^**  **(38.09 ng/ml)** | ***P* value** |
| --- | --- | --- | --- | --- |
| N | 20 | 703 | 20 |  |
| Age, y | 52.0±13.3 | 47.8±11.9 | 47.5±13.2 | 0.306 |
| Smoking, n (%) | 0 (0) | 7 (1.0) | 0 (0) | 0.818 |
| Hypertension, n (%) | 0 (0) | 121 (17.2) | 2 (10.0) | 0.158 |
| Diabetes, n (%) | 3 (15.0) | 54 (7.7) | 2 (10.0) | 0.462 |
| Dyslipidemia, n (%) | 2 (10) | 77 (11.0) | 4 (20.0) | 0.656 |
| BMI, kg/m^2^* | 21.4±1.1 | 23.0±1.1 | 23.5±1.4 | <0.001 |
| WC, cm* | 70.1±5.0 | 76.7±5.8 | 79.8±6.3 | <0.001 |
| WC ≥85 cm, n (%)* | 0 (0) | 60 (8.5) | 5 (25) | 0.014 |
| HC, cm* | 89.8±4.6 | 93.6±4.2 | 95.6±5.4 | <0.001 |
| SBP, mmHg | 121.3±17.2 | 123.4±18.4 | 123.2±17.2 | 0.885 |
| DBP, mmHg | 69.8±9.4 | 73.5±10.2 | 75.4±7.7 | 0.190 |
| FPG, mmol/L | 6.11±2.59 | 5.67±1.27 | 6.09±2.03 | 0.144 |
| HOMA-IR, median (25^th^, 75^th^)* | 0.93 (0.66, 2.13) | 1.63 (1.14, 2.27) | 2.28 (1.71, 4.15) | <0.001 |
| 2h-PPG, mmol/L | 6.60±1.81 | 6.73±1.94 | 7.49±2.42 | 0.248 |
| HbA1c, % | 5.91±1.46 | 5.65±0.72 | 5.56±0.67 | 0.251 |
| TC, mmol/L | 4.70±0.82 | 4.84±0.95 | 4.93±0.69 | 0.730 |
| TG, mmol/L | 0.74±0.43 | 1.11±0.93 | 1.33±0.89 | 0.106 |
| HDL-C, mmol/L | 1.43±0.21 | 1.29±0.30 | 1.24±0.26 | 0.093 |
| LDL-C, mmol/L | 2.66±0.75 | 2.81±0.78 | 2.90±0.42 | 0.591 |
| ALT, U/L | 17.9±7.6 | 18.3±11.3 | 19.5±6.10 | 0.892 |
| AST, U/L | 21.4±8.1 | 21.3±8.1 | 21.7±6.4 | 0.969 |
| Serum Cr, μmol/L* | 52.0±12.4 | 50.9±8.9 | 72.3±8.2 | <0.001 |
| UA, μmol/L* | 208.3±45.5 | 232.6±55.8 | 264.4±55.7 | 0.006 |
| MetS, n (%) | 4 (20.0) | 238 (33.9) | 6 (30.0) | 0.410 |
| Leptin, ng/ml* | 2.93±0.87 | 16.25±7.43 | 47.54±12.11 | <0.001 |

Continuous variables were expressed as mean ± standard deviation or median (25%th, 75%th), and categorical variables were expressed as n (%).

* P values of the difference between the leptin >97.5^th^ group and the leptin of 2.5^th^ to ≤97.5^th^ group, and the difference between the leptin >97.5^th^ group and the leptin <2.5^th^ group were <0.025.

**Table S4** Clinical characteristics of men with BMI of 20 to <25 by tertiles of leptin levels

|  | **First tertile (leptin ≤2.04 ng/ml)** | **Second tertile (leptin of 2.04 to ≤ 4.39 ng/ml)** | **Third tertile (leptin >4.39 ng/ml)** | ***P* value** |
| --- | --- | --- | --- | --- |
| N | 218 | 220 | 217 |  |
| Age, y | 52.9±10.9 | 52.3±10.5 | 50.8±13.6 | 0.147 |
| Smoking, n (%)* | 168 (77.1) | 146 (66.4) | 133 (61.3) | 0.001 |
| Hypertension, n (%)* | 29 (13.3) | 52(23.6) | 57 (26.3) | 0.009 |
| Diabetes, n (%)* | 7 (3.2) | 17 (7.7) | 27 (12.4) | 0.002 |
| Dyslipidemia, n (%)* | 10 (4.6) | 28 (12.7) | 48 (22.1) | <0.001 |
| BMI, kg/m^2^* | 22.3±1.4 | 23.1±1.2 | 23.5±1.2 | <0.001 |
| WC, cm* | 77.8±5.1 | 81.2±4.7 | 84.8±5.4 | <0.001 |
| WC ≥90cm, n (%)* | 1 (0.5) | 8 (3.6) | 37 (17.1) | <0.001 |
| HC, cm* | 92.1±5.1 | 94.4±4.6 | 95.1±4.3 | <0.001 |
| SBP, mmHg | 131.8±18.7 | 131.7±17.9 | 128.2±15.8 | 0.055 |
| DBP, mmHg | 77.7±10.4 | 78.3±10.6 | 78.2±11.3 | 0.808 |
| FPG, mmol/L | 5.8±1.4 | 6.1±1.7 | 6.2±1.8 | 0.092 |
| HOMA-IR, median (25^th^, 75^th^)* | 1.00 (0.74, 1.32) | 1.29 (0.99, 1.80) | 1.94 (1.32, 2.87) | <0.001 |
| 2h-PPG, mmol/L | 6.7±2.9 | 6.9±2.3 | 7.1±2.4 | 0.243 |
| HbA1c, % | 5.6±0.9 | 5.7±1.1 | 5.8±1.0 | 0.199 |
| TC, mmol/L | 4.78±0.96 | 4.84±0.94 | 4.93±0.94 | 0.218 |
| TG, mmol/L* | 0.90±0.57 | 1.29±1.23 | 1.79±1.58 | <0.001 |
| HDL-C, mmol/L* | 1.36±0.42 | 1.23±0.33 | 1.09±0.27 | <0.001 |
| LDL-C, mmol/L* | 2.69±0.72 | 2.77±0.73 | 2.94±0.80 | 0.002 |
| ALT, U/L* | 20±11 | 21±9 | 24±12 | <0.001 |
| AST, U/L | 25±15 | 23±8 | 23±7 | 0.069 |
| Serum Cr, μmol/L* | 66±10 | 67±10 | 72±13 | <0.001 |
| UA, μmol/L* | 278±64 | 295±57 | 332±77 | <0.001 |
| MetS, n (%)* | 47 (21.6) | 90 (40.9) | 113 (52.1) | <0.001 |
| Leptin, ng/ml* | 1.19±0.51 | 3.15±0.65 | 7.87±3.35 | <0.001 |

Continuous variables were expressed as mean ± standard deviation or median (25%th, 75%th), and categorical variables were expressed as n (%).

* P values of the difference between the third tertile group and the second tertile group, and the difference between the third tertile group and the first tertile group were <0.025.

**Table S5** Clinical characteristics of women with BMI of 20 to <25 by tertiles of leptin levels

|  | **First tertile (leptin ≤11.98 ng/ml)** | **Second tertile (leptin of 11.98 to ≤ 18.78 ng/ml)** | **Third tertile (leptin >18.78 ng/ml)** | ***P* value** |
| --- | --- | --- | --- | --- |
| N | 247 | 248 | 248 |  |
| Age, y* | 49.7±12.2 | 47.4±12.0 | 46.6±11.6 | 0.010 |
| Smoking, n (%) | 3 (1.2) | 3 (1.2) | 1 (0.4) | 0.560 |
| Hypertension, n (%) | 44 (17.8) | 41 (16.5) | 38 (15.3) | 0.863 |
| Diabetes, n (%) | 19 (7.7) | 24 (9.7) | 16 (6.5) | 0.407 |
| Dyslipidemia, n (%) | 24 (9.7) | 28 (11.3) | 31 (12.5) | 0.063 |
| BMI, kg/m^2^* | 22.4±1.3 | 23.1±1.3 | 23.4±1.2 | <0.001 |
| WC, cm* | 74.6±5.9 | 77.2±5.8 | 78.0±5.6 | <0.001 |
| WC ≥85 cm, n (%)* | 11 (4.5) | 24 (9.7) | 30 (12.1) | 0.009 |
| HC, cm* | 92.5±4.2 | 93.6±4.3 | 94.6±4.1 | <0.001 |
| SBP, mmHg | 125.2±19.1 | 123.9±18.5 | 120.9±17.1 | 0.031 |
| DBP, mmHg | 73.3±10.5 | 73.6±10.4 | 73.3±9.6 | 0.932 |
| FPG, mmol/L | 5.6±1.3 | 5.7±1.4 | 5.7±1.3 | 0.783 |
| HOMA-IR, median (25^th^, 75^th^)* | 1.28 (0.91, 1.80) | 1.61 (1.20, 2.26) | 1.97 (1.52, 2.95) | <0.001 |
| 2h-PPG, mmol/L* | 6.6±2.1 | 6.6±1.7 | 7.0±2.0 | 0.016 |
| HbA1c, % | 5.6±0.7 | 5.7±0.8 | 5.7±0.7 | 0.853 |
| TC, mmol/L | 4.77±0.90 | 4.87±0.91 | 4.88±1.01 | 0.378 |
| TG, mmol/L* | 0.86±0.47 | 1.22±1.24 | 1.25±0.84 | <0.001 |
| HDL-C, mmol/L | 1.33±0.30 | 1.28±0.28 | 1.27±0.31 | 0.025 |
| LDL-C, mmol/L | 2.74±0.77 | 2.83±0.77 | 2.84±0.77 | 0.279 |
| ALT, U/L | 18±14 | 18±9 | 18±10 | 0.991 |
| AST, U/L | 22±9 | 21±6 | 21±8 | 0.642 |
| Serum Cr, μmol/L | 50±8 | 51±9 | 54±25 | 0.036 |
| UA, μmol/L* | 219±52 | 236±54 | 243±58 | <0.001 |
| MetS, n (%) | 75 (30.4) | 83 (33.5) | 90 (36.3) | 0.376 |
| Leptin, ng/ml* | 8.13±2.56 | 15.18±1.84 | 26.85±8.50 | <0.001 |

Continuous variables were expressed as mean ± standard deviation or median (25%th, 75%th), and categorical variables were expressed as n (%).

* P values of the difference between the third tertile group and the second tertile group, and the difference between the third tertile group and the first tertile group were <0.025.

**Table S6** ROC analysis for determining cut-off point of serum leptin levels (ng/ml) to distinguish the participants with BMI <25 kg/m^2^ (normal weight) and BMI ≥25 kg/m^2^ (overweight or obesity)

| **Sex** | **Cut-off point** | **Sensitivity, % (95%CI)** | **Specificity, % (95%CI)** | **Area under curve (AUC)** | ***P* value** |
| --- | --- | --- | --- | --- | --- |
| Men (n=469) | 4.65 | 81.0 (75.4, 85.8) | 75.0 (68.9, 80.4) | 0.847 | <0.001 |
| Women (n=773) | 19.69 | 73.8 (68.6, 78.6) | 75.9 (71.7, 79.7) | 0.817 | <0.001 |
